# Applicability of artificial intelligence-based computer-aided detection (AI–CAD) for pulmonary tuberculosis to community-based active case finding

**DOI:** 10.1186/s41182-023-00560-6

**Published:** 2024-01-02

**Authors:** Kosuke Okada, Norio Yamada, Kiyoko Takayanagi, Yuta Hiasa, Yoshiro Kitamura, Yutaka Hoshino, Susumu Hirao, Takashi Yoshiyama, Ikushi Onozaki, Seiya Kato

**Affiliations:** 1 The Research Institute of Tuberculosis (RIT), Japan Anti-Tuberculosis Association (JATA), Tokyo, Japan; 2 Department of International Programme, Japan Anti-Tuberculosis Association (JATA), Tokyo, Japan; 3 Fukujuji Hospital, Japan Anti-Tuberculosis Association (JATA), Tokyo, Japan; 4 Imaging Technology Center, ICT Strategy Division, Fujifilm Corporation, Tokyo, Japan

**Keywords:** Pulmonary tuberculosis, Artificial intelligence, Computer-aided detection, Active case finding, Ultra-portable CXR, CXR screening

## Abstract

**Background:**

Artificial intelligence-based computer-aided detection (AI–CAD) for tuberculosis (TB) has become commercially available and several studies have been conducted to evaluate the performance of AI–CAD for pulmonary tuberculosis (TB) in clinical settings. However, little is known about its applicability to community-based active case-finding (ACF) for TB.

**Methods:**

We analysed an anonymized data set obtained from a community-based ACF in Cambodia, targeting persons aged 55 years or over, persons with any TB symptoms, such as chronic cough, and persons at risk of TB, including household contacts. All of the participants in the ACF were screened by chest radiography (CXR) by Cambodian doctors, followed by Xpert test when they were eligible for sputum examination. Interpretation by an experienced chest physician and abnormality scoring by a newly developed AI–CAD were retrospectively conducted for the CXR images. With a reference of Xpert-positive TB or human interpretations, receiver operating characteristic (ROC) curves were drawn to evaluate the AI–CAD performance by area under the ROC curve (AUROC). In addition, its applicability to community-based ACFs in Cambodia was examined.

**Results:**

TB scores of the AI–CAD were significantly associated with the CXR classifications as indicated by the severity of TB disease, and its AUROC as the bacteriological reference was 0.86 (95% confidence interval 0.83–0.89). Using a threshold for triage purposes, the human reading and bacteriological examination needed fell to 21% and 15%, respectively, detecting 95% of Xpert-positive TB in ACF. For screening purposes, we could detect 98% of Xpert-positive TB cases.

**Conclusions:**

AI–CAD is applicable to community-based ACF in high TB burden settings, where experienced human readers for CXR images are scarce. The use of AI–CAD in developing countries has the potential to expand CXR screening in community-based ACFs, with a substantial decrease in the workload on human readers and laboratory labour. Further studies are needed to generalize the results to other countries by increasing the sample size and comparing the AI–CAD performance with that of more human readers.

## Introduction

Tuberculosis (TB) is a health-threatening infectious disease, with an estimated 10.6 million incident cases and 1.6 million deaths, including HIV-positive people per year worldwide [1]. To tackle this disease, the World Health Organization (WHO) announced the End TB Strategy [2] which defines targets to reduce incidence by 90% and TB deaths by 95% by 2035. In addition, the United Nations General Assembly held its first-ever high-level meeting on TB in 2018, and adopted a political declaration of 40 million treatments and 30 million TB preventive treatments for 5 years for an urgent global response to a global epidemic [3]. Therefore, implementation of evidence-based strategies that can lead to early case detection, proper treatment, and resulting reduction of TB transmission is required.

Despite these global efforts, there was a decrease of 18% from 7.1 million to 5.8 million in case notifications between 2019 and 2020 because of the COVID-19 pandemic, and a partial recovery to 6.4 million in 2021, while the declining trends of TB deaths since 2005 inversely increased to the level of 2017 [1]. Therefore, actions to mitigate and reverse the impact of the COVID-19 pandemic on TB are urgently needed.

Chest radiography (CXR) for the diagnosis or screening of pulmonary TB has been limited by modest specificity, high inter- and intrareader differences in interpretation, and suboptimal quality images in many developing countries [4, 5]. Bacteriological examinations for sputum have been recommended, as represented by the directly observed treatment, short-course (DOTS) strategy [6]. After the first national TB prevalence survey in Cambodia [7], where CXR was used for screening purposes for eligibility for sputum examination, prevalence surveys in a standardised manner were carried out in many countries with high TB burdens to measure TB prevalence [8–10]. As a result, more attention has been given to both the role of CXR in TB case detection, and the concept of subclinical TB, i.e., the presence of persons without typical TB symptoms in the community [11, 12]. At the same time, researchers have begun to think of TB case detection by active case-finding (ACF), in which usually asymptomatic persons at high risk are actively screened for TB, as well as passive case-finding (PCF), in which symptomatic persons seek health care by themselves. However, the challenges in community-based ACF include the consumption of resources, such as labour and medical equipment, the requirement of large sample sizes, and its high cost, although it might be effective in changing TB epidemiology [13]. Thus, the WHO published Systematic screening for active TB in 2013 [14], and Consolidated guidelines on TB in 2021 [15], which specifies targeted groups for ACF, and screening tools to be used, including artificial intelligence-based computer-aided detection (AI–CAD).

During this period, remarkable progress in CXR equipment was made. Replacing analogue images that needed film processing, computed radiography, and digital radiography, which enabled us to check an image soon after shooting, has been rapidly rolled out in developing countries. The digitalization of radiology has solved technical challenges, such as manual film processing, reagent replacement, and maintenance of film processors, and has brought high-quality images of CXR even in resource-limited settings. Furthermore, a handy CXR equipment called ultra-portable CXR [16] has made TB screening possible in communities located far from medical facilities. The development of AI–CAD has paved the way to solve another major challenge: the interpretation of CXR images due to shortages of radiologists or chest physicians in developing countries.

Several studies on the performance of AI–CAD for TB [17–21] have shown that it is comparable to or better than that of experienced medical doctors. However, these studies were mostly conducted for people with TB detected in PCF, and little is known about its applicability to community-based ACF for pulmonary TB [22–24].

Therefore, we examined the performance of a deep learning algorithm for TB detection (F-CAD) developed as a prototype by FUJIFILM Corporation, and its applicability using a data set obtained from a community-based ACF in Cambodia, a country with a high TB burden.

## Methods

### Development of a deep learning algorithm for TB detection

#### For F-CAD training

We formed a training set and a parameter tuning set by patientwise splitting of the following data sets: 1464 CXRs with positive molecular tests for TB, 2914 CXRs with active TB in the radiology report, 3139 CXRs with other abnormalities but negative TB in the radiology report and 6350 normal CXRs retrospectively collected from two diagnostic centers in India, and 60,326 CXRs with other abnormalities, such as atelectasis, pleural effusion, or fibrosis pattern and 37,716 normal CXRs from the PadChest data set [25].

#### Development of the algorithm

The proposed AI–CAD consists of a two-stage pipeline. In the first stage, we segmented the lung and heart regions using a U-Net model [26] for the intensity and spatial normalization of an input CXR. We adjusted the mean and standard deviations of pixel values in the lung region to 0 and 1, respectively. Then, it was trimmed by the circumscribed rectangle of the lung and heart regions to reduce the variability in surrounding objects.

In the second stage, given a normalized CXR, a classification score and a localization map were predicted using a CNN (convolutional neural network) model, which was the cascade of a DenseNet feature extractor [27] and a pixelwise localizer. The localizer consisted of a convolutional layer and a global maximum pooling layer. Of note, the outputs from the two layers corresponded to the classification score and the localization map. Both the score and the localization map had values between 0 and 1, representing the probabilities of any active TB findings. In the training phase, the model was optimized using only image-level annotation and an entropy-based loss function. Data augmentation techniques, such as random resizing, cropping, horizontal flipping, rotation, Gaussian noise, and salt-and-pepper noise were applied to enhance the generalization performance. We applied energy spectrum modification and grid artefact injection to improve the robustness against software postprocessing algorithms, such as dynamic compression and hardware failure. At the inference phase, the outputs from the three models being trained using three different hyperparameters with test-time augmentation [28] were aggregated by averaging to produce the final prediction. For internal validation using two TB data sets publicly available from the National Library of Medicine [29], the area under the receiver operating characteristic (ROC) curve (AUROC) was 0.969 and 0.996 on the Shenzhen and Montgomery data sets, respectively.

### Preparation of data set

#### Active case finding in Cambodia

The Cambodia Anti-Tuberculosis Association (CATA) conducted community-based ACFs for 88,316 participants in 32 operational districts from November 2018 until November 2021. It targeted all persons aged 55 years or older; persons with TB-related symptoms, such as cough, fever, and night sweats for more than 2 weeks; and persons at risk of TB, such as diabetes mellitus, household contacts, and past TB history [30]. All participants excluding pregnant women and refusers were screened by a digital CXR. A doctor working for CATA screened the participants based on their CXR results: “normal”, “active TB”, “suspect TB”, “healed TB”, and “other lung diseases”. If the participant had a CXR suggestive of “active TB”, “suspect TB”, or sometimes “healed TB”, a sputum specimen was taken on the spot for Xpert testing, which was performed on the ground by a mobile team. The participants’ data, Xpert test results, and CXRs in the form of DICOM (digital imaging and communications in medicine) images were stored and strictly managed in computers.

#### Data selection and anonymisation

We prepared 8,519 CXR images and medical data of participants in the ACFs at 13 districts randomly selected on a district basis from the 32 districts due to the suboptimal management of data storage, and anonymized them with an identifier number for analysis.

#### AI–CAD analysis and human reading by chest physician

Interpretation of the images by a chest physician who had more than 10 years of experience, and TB scoring by F-CAD were retrospectively conducted. In doing so, neither the F-CAD developer nor the human reader was informed of the results of Xpert or CXR interpretation in Cambodia, and both were blinded to each other’s results. TB scores were provided by a continuous number between 0 and 1, which were more generally suggestive of active TB when larger. The interpretations by the physician basically followed the five classifications in Cambodia, but “active TB” was further classified into two categories: “active TB with cavity” and “active TB without cavity”.

#### Performance evaluation and statistical analysis

To evaluate F-CAD performance, we used ROC curves and AUROCs [31] as the bacteriological reference of the Xpert results, and the radiological reference by human readings. In the analysis, “active TB with and without cavity” was defined as “abnormality strongly suggestive of TB”; “active TB and suspect TB” as “abnormality suggestive of TB”; “active TB, suspect TB and healed TB” as “abnormality suggestive of any TB”; and “active TB, suspect TB, healed TB and other lung diseases” as “any abnormality in lung fields”. The ROC curves were made for triage purposes in which possibly infectious cases are effectively selected with the reference of “abnormality suggestive of TB”, and for screening purposes in which suspected TB cases are widely selected with the reference of “any abnormality in lung fields”. The AUCs were calculated using the pROC package of R version 4.1.2 (R Foundation for Statistical Computing, Vienna, Austria), and DeLong methods [32]. We also examined precision–recall curves (PRCs) [33], because the data set was imbalanced with a low Xpert positivity rate.

In addition, we examined whether F-CAD meets the target product profile (TPP) by the WHO [34]: 90% sensitivity/70% specificity as minimal requirements, and 95% sensitivity/80% specificity as optimal requirements, and its applicability to community-based ACF in Cambodia using the data set in the study.

We presented TB scores as the medians with interquartile ranges (IQRs) and used Mann–Whitney’s *U* test as a statistical test. The developer of F-CAD was not part of the study team and had no role in the study design, data collection, analysis, or interpretation of the results. This study was approved by the National Ethics Committee for Health Research, Cambodia.

## Results

### Demographic and clinical characteristics of persons screened with chest X-ray

We analysed the final data set of 8,386 CXRs and medical data after excluding 133 CXRs because of 59 duplications and 74 without any matching medical data. Table 1 shows the demographic and clinical characteristics of persons screened with CXR; 5,584 (67% of the participants) were female, and 2,839 (34%) were 65 years or older. A total of 5,202 (62%) had a cough for more than 2 weeks. The percentages of the participants with diabetes mellitus, positive HIV status, and smoking as TB risk were 5.3%, 0.5%, and 14%, respectively. A total of 1,145 (14%) had contacts of TB, and 993 (12%) had a past history of TB. Overall, 1,371 (16%) were examined by Xpert on the ground. Of them, 130 (1.6% of the participants and 9.5% of the persons examined by Xpert) were positive for Xpert.

**Table 1:** Demographic and clinical characteristics of persons screed with chest X-ray

	Xpert test			Total
	Positive (%)	Negative (%)	Not examined	
N	130 (1.6)	1241 (14.8)	7015 (83.7)	8386 (100)
Sex				
Male	65 (2.3)	517 (18.5)	2220 (79.2)	2802 (100)
Female	65 (1.2)	724 (13.0)	4795 (85.9)	5584 (100)
Age				
15–24	4 (2.0)	6 (3.0)	189 (95.0)	199 (100)
25–34	6 (1.4)	20 (4.5)	415 (94.1)	441 (100)
35–44	18 (2.2)	52 (6.4)	742 (91.4)	812 (100)
45–54	22 (1.4)	165 (10.8)	1339 (87.7)	1526 (100)
55–64	27 (1.1)	361 (14.3)	2133 (84.6)	2521 (100)
65-	53 (1.9)	634 (22.3)	2152 (75.8)	2839 (100)
Unknown	0 (0.0)	3 (6.3)	45 (93.8)	48 (100)
TB symptoms				
Cough (yes)	108 (2.1)	908 (17.5)	4186 (80.5)	5202 (100)
(No)	22 (0.7)	333 (10.5)	2829 (88.9)	3184 (100)
Fever (yes)	86 (1.9)	758 (16.3)	3800 (81.8)	4644 (100)
(No)	44 (1.2)	483 (12.9)	3215 (85.9)	3742 (100)
Night sweat (yes)	54 (1.3)	621 (15.2)	3402 (83.4)	4077 (100)
(No)	76 (1.8)	620 (14.4)	3613 (83.8)	4309 (100)
Weight loss (yes)	79 (2.2)	636 (17.5)	2919 (80.3)	3634 (100)
(No)	51 (1.1)	605 (12.7)	4096 (86.2)	4752 (100)
Lymph node swelling (yes)	4 (1.5)	26 (9.7)	239 (88.8)	269 (100)
(No)	126 (1.6)	1215 (15.0)	6776 (83.5)	8117 (100)
Other risk factors				
Diabetes mellitus (yes)	9 (2.0)	64 (14.4)	372 (83.6)	445 (100)
(No)	121 (1.5)	1177 (14.8)	6643 (83.7)	7941 (100)
HIV (yes)	0 (0.0)	12 (30.8)	27 (69.2)	39 (100)
(No or unknown)	130 (1.6)	1229 (14.7)	6988 (83.7)	8347 (100)
Smoking (yes)	22 (1.9)	200 (16.8)	965 (81.3)	1187 (100)
(No)	108 (1.5)	1041 (14.5)	6050 (84.0)	7199 (100)
Family TB history (yes)	13 (1.4)	127 (14.0)	764 (84.5)	904 (100)
(No)	117 (1.6)	1114 (14.9)	6251 (83.5)	7482 (100)
TB contact (yes)	17 (1.5)	141 (12.3)	987 (86.2)	1145 (100)
(No)	113 (1.6)	1100 (15.2)	6028 (83.2)	7241 (100)
Past TB history (yes)	15 (1.5)	299 (30.1)	679 (68.4)	993 (100)
(No)	115 (1.6)	942 (12.7)	6336 (85.7)	7393 (100)

### Chest X-ray reading and Xpert results

The results of human reading by the chest physician indicated many abnormal findings on CXR, as shown in Table 2, probably reflecting a past epidemic of TB in Cambodia and the participation of elderly individuals: 6,835 (82%) with normal CXR, 414 (5%) with active TB, 201 (2%) with suspected TB, 841 (10%) with healed TB, and 95 (1%) with other lung diseases. Of the “active TB” individuals, positive Xpert, negative Xpert, and not performed Xpert were 24%, 43%, and 33%, respectively. Of the “suspect TB”, positive Xpert, negative Xpert, and not performed Xpert were 5%, 49%, and 46%, respectively. There were 17 Xpert-positive cases in “healed TB” and 3 in “other lung diseases”. No rifampicin-resistant TB was detected among the Xpert-positive TB cases.

**Table 2:** CXR reading by human reader and Xpert results

Results of CXR reading	Xpert			Total
	Positive	Negative	Not examined	
Normal lung field	1 (0.0)	568 (8.3)	6266 (91.7)	6835 (100)
Active TB	99 (23.9)	179 (43.2)	136 (32.9)	414 (100)
Suspect TB	10 (5.0)	99 (49.3)	92 (45.8)	201 (100)
Healed TB	17 (2.0)	346 (41.1)	478 (56.8)	841 (100)
Other lung disease	3 (3.2)	49 (51.6)	43 (45.3)	95 (100)
Total	130 (1.6)	1241 (14.8)	7015 (83.7)	8386 (100)

### Results of human reader and TB scores

**Fig. 1:**
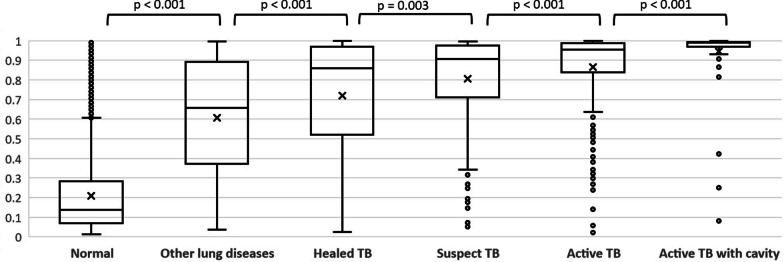
Interquartile ranges of TB scores by classification of human readings for chest X-ray

The IQR of TB scores by classification of human readings for CXR are shown in Fig. 1. The TB scores of F-CAD were significantly associated with the results of the human reader for CXR as indicated by the severity of TB disease: the median of “active TB with cavity”, “active TB without cavity”, “suspect TB”, “healed TB”, “other lung disease”, and “normal” was 0.99, 0.95, 0.91, 0.86, 0.66, and 0.14, respectively.

### Performance with the bacteriological reference by Xpert results

**Fig. 2:**
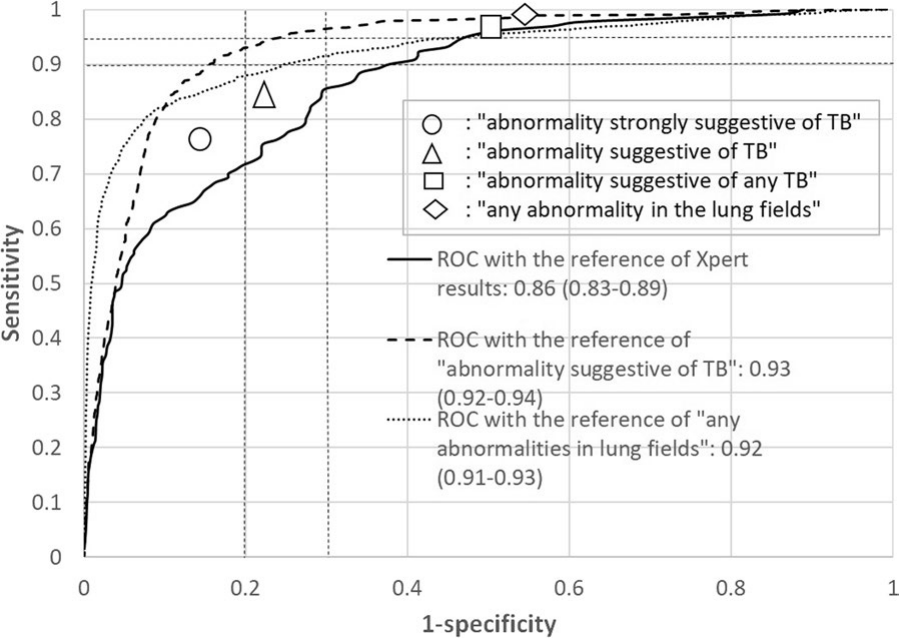
Performance of F-CAD and human reading

The ROC curve of TB scores with the bacteriological reference is shown in Fig. 2, as well as the sensitivities and specificities based on the classification by the human reader. The AUROC of F-CAD was 0.86 (95% confidence interval (CI) 0.83–0.89). When we compared the AUROC by age group, the AUROC for those aged 65 or older was significantly lower [0.80 (95% CI 0.73–0.80)] than that for those aged under 65 years [0.91 (95% CI 0.88–0.91)], although it was not shown in the figure. We plotted the sensitivities and specificities by the human reader based on “abnormality strongly suggestive of TB”, “abnormality suggestive of TB”, “abnormality suggestive of any TB”, and “any abnormality in lung fields” with 76%/85%, 84%/78%, 97%/50%, and 99%/46%, respectively.

**Fig. 3:**
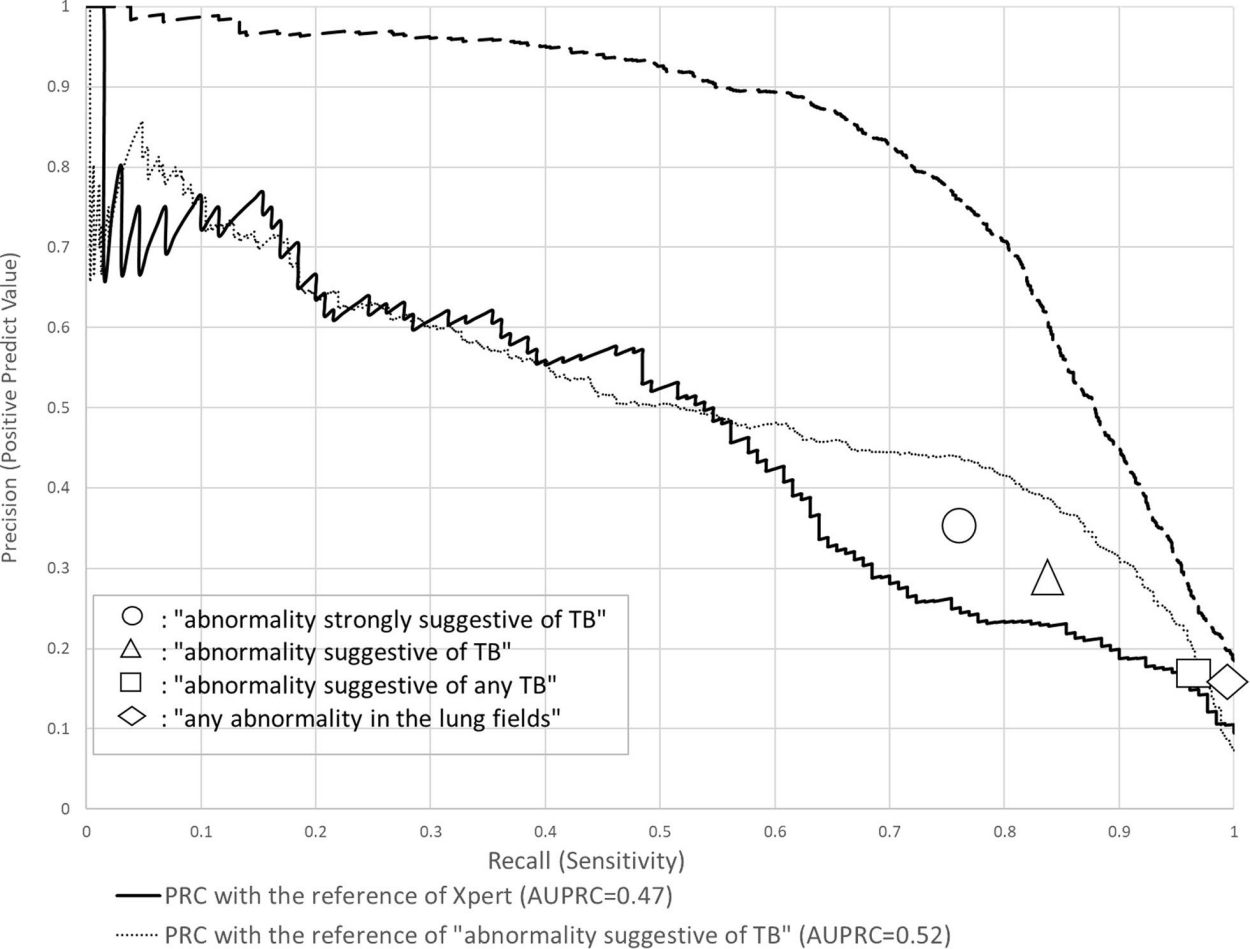
Precision Recall curve for F-CAD and human reading

Figure 3 shows the PRC curves of TB scores with the bacteriological reference. It declined in a linear manner as the sensitivity increased, and reached a positive predictive value (PPV) of 0.1, which was obtained from 130 divided by 1,371 as the lowest PPV. The area under the PRC (AUPRC) was 0.47. Sensitivities and PPVs by the human reader were 76%/36% for “abnormality strongly suggestive of TB”, 84%/28% for “abnormality suggestive of TB”, 97%/17% for “abnormality suggestive of any TB”, and 99%/16% for “any abnormality in lung fields”.

### Performance with the radiological reference by human readings

The AUROCs with the reference of “abnormality suggestive of TB” as a triage purpose, and with the reference of “any abnormality in the lung fields” as a screening purpose were 0.93 (95% CI 0.92–0.94), and 0.92 (95% CI 0.91–0.93), respectively, as shown in Fig. 2. The AUPRCs with the reference of “abnormality suggestive of TB” and with the reference of “any abnormality in the lung fields” shown in Fig. 3 were 0.52 and 0.83, respectively. The PPVs at 90% sensitivity were nearly 30% for triage purposes, and more than 40% for screening purposes.

### Performance against WHO’s target product profile by reference

The performance against the WHO’s TPP by reference is shown in Table 3. With the bacteriological reference, no sensitivity or specificity met the WHO’s TPP: 62% (95% CI 0.59–0.65) specificity at 90% sensitivity, and 85% (95% CI 0.85–0.91) sensitivity at 70% specificity. With the radiological reference of “abnormality suggestive of TB”, however, the corresponding sensitivities or specificities reached the targets: 84% (95% CI 0.84–0.85) specificity at 90% sensitivity and 96% (95% CI 0.95–0.98) sensitivity at 70% specificity. With the radiological reference of “any abnormality in the lung fields”, 75% (95% CI 0.74–0.76) specificity at 90% sensitivity reached the target; however, the lower margin of 91% (95% CI 0.90–0.93) sensitivity at 70% specificity did not exceed 90%.

**Table 3:** Performance of F-CAD against WHO's Target Product Profile by reference

Reference		Xpert results	"abnormality suggestive of TB"	"any abnormality in the lung fields"
Sensitivity≧95	Actual sensitivity	0.954 (0.902–0.983)	0.951 (0.931–0.967)	0.950 (0.938–0.961)
	TB score	0.72	0.36	0.15
	Specificity (95%CI)	0.517 (0.488–0.545)	0.747 (0.737–0.757)	0.525 (0.513–0.537)
Sensitivity≧90	Actual sensitivity	0.900 (0.835–0.946)	0.901 (0.874–0.923)	0.900 (0.884–0.915)
	TB score	0.88	0.53	0.28
	Specificity (95%CI)	0.621 (0.594–0.648)	0.844 (0.835–0.852)*	0.750 (0.739–0.760)*
Specificity≧80	Actual specificity	0.800 (0.777–0.822)	0.800 (0.791–0.809)	0.800 (0.790–0.809)
	TB score	0.97	0.44	0.34
	Sensitivity (95%CI)	0.715 (0.630–0.791)	0.930 (0.907–0.949)	0.879 (0.862–0.895)
Specificity≧70	Actual specificity	0.700 (0.674–0.726)	0.700 (0.690–0.710)	0.700 (0.689–0.711)
	TB score	0.93	0.31	0.25
	Sensitivity (95%CI)	0.854 (0.854–0.910)	0.964 (0.946–0.977)*	0.914 (0.899–0.928)

*Significantly over Target Product Profile

### Performance set at 90% sensitivity for community-based ACF

**Fig. 4:**
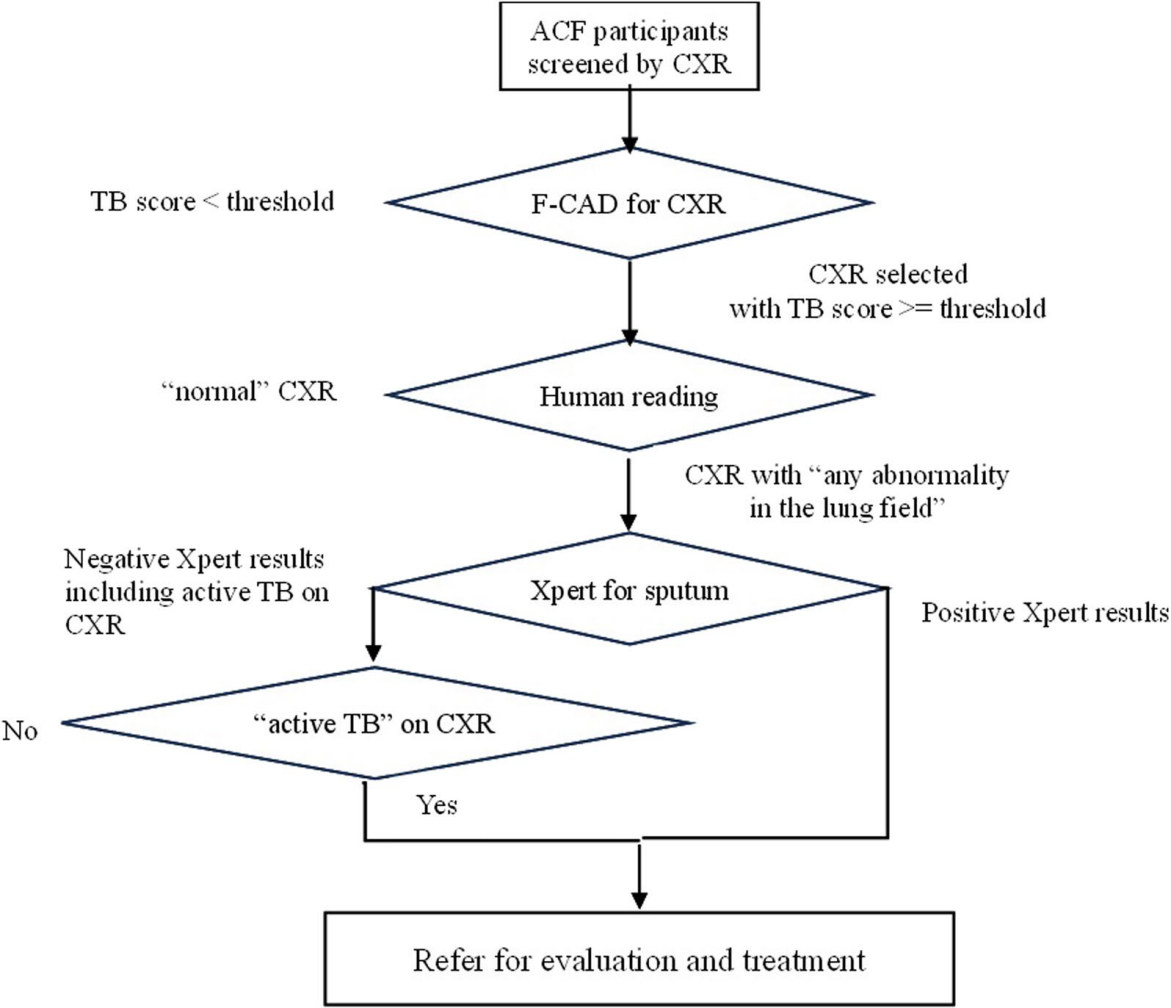
Proposed algorithm for community-based ACF

Given that we used a TB score by F-CAD as the threshold for triage purposes or screening purposes set at 90% sensitivity, we examined how many CXRs by human reader and bacteriological examinations could be decreased in number, and how many cases with positive Xpert, with “abnormality suggestive of TB”, and with “any abnormality in the lung fields” could be missed using F-CAD. A proposed algorithm for community-based ACF using both F-CAD and human reading in combination with Xpert test is shown in Fig. 4. As shown in Table 4, if we used a threshold of 0.5340 for triage purposes, the bacteriological examinations fell to 15% of the original number by the reduction to 21% of CXR to be interpreted, followed by the exclusion of 524 normal CXRs by human reading. On the other hand, TB cases detected as “abnormality suggestive of TB” and positive Xpert could be maintained at 90% and 96%, respectively. Similarly, if we used a threshold of 0.2835 for screening purposes, we could maintain TB cases detected as “abnormality suggestive of TB” and positive Xpert at 97% and 98%, respectively, while the bacteriological examinations fell to 17% in number by the reduction of CXRs by human reading to 37%, followed by the exclusion of the cases with normal CXRs.

**Table 4:** Performance in triage purposes and screening purposes at sensitivity of 90%

Purpose	TB score as threshold	N of CXR selected (% of 8,386)	N of sputum exams* (% of 8,386)	N of Xpert-positive (% of 130)	N of CXR with "abnormality suggestive of TB" (% of 615)	N of CXR with "any abnormality in the lung fields" (% of 1551)
Triage	0.5340	1770 (21.1)	1246 (14.9)	125 (96.2)	554 (90.1)	1246 (80.3)
Screening	0.2835	3107 (37.0)	1396 (16.6)	127 (97.7)	594 (96.6)	1396 (90.0)

*N of sputum exams = N of CXR selected—N of normal CXR by human reading

## Discussion

TB scores of F-CAD were significantly associated with the CXR classifications as indicated by the severity of TB disease. The AUROC as the bacteriological reference was 0.86 (95% CI 0.83–0.89), which was similar to 0.82–0.94 in other recent studies on the best 3 AI–CAD algorithms [19–21]. When we used a threshold for triage purposes at 90% sensitivity as the radiological reference, human readings and bacteriological examinations needed fell to 21% and 15%, respectively, maintaining 95% of Xpert-positive TB to be detected in ACF. Similarly, for screening purposes, we could maintain 98% of Xpert-positive TB. The study suggested that the use of AI–CAD in developing countries has the potential to expand CXR screening for TB in community-based ACFs with a substantial decrease in the workload on human readers and laboratory labour.

Despite any effort to combat TB across the globe, an annual reduction in TB incidence rate before the COVID-19 pandemic was only 2.3% between 2018 and 2019 [35], and the acceleration of reduction has been required towards ending TB. A recent study showed that systematic screening for TB based on symptom screening alone may not be sufficient to achieve a large reduction in TB prevalence over a period of a few years [36]. The active use of CXR equipped with AI–CAD in high TB burden countries can be a key to improving detection of cases with asymptomatic, subclinical TB as well as symptomatic TB.

The F-CAD system can work on a laptop computer without an internet connection, and an ultra-portable CXR system, including a digital panel for X-ray detection can be operated by battery power in the field.

One of the strengths of the study is the use of real data obtained from community-based ACF for general people in Cambodia with a high burden of TB. The study subjects were more likely to be asymptomatic or to have milder symptoms and more normal CXRs than those in clinical settings [37]. Most studies conducted on AI–CAD evaluation [17–21] used medical data from hospital patients with severe symptoms and high TB prevalence, and there are only a few studies for general populations in the community [22–24]. In addition, the AUROCs of F-CAD with the bacteriological reference were comparable to other CAD algorithms, and therefore, the conclusion from the study on the potentiality of AI–CAD use for community-based ACF is plausible, although further comparative studies are needed.

There are several limitations in the study. First, the quality of CXR images was challenging, because the shooting conditions might not have always been properly set. Some images had artefacts with belt-shaped patterns of stripes with light and shade, which might have affected the TB scores and human readings of the results. Second, because we used data obtained from the actual ACF for TB in the community, their bacteriological examinations were limited to 16% of the participants who had been screened by CXR, and a few persons with positive Xpert might have been missed. However, we believe that the performance of AI–CAD should be evaluated using both radiological and bacteriological references, because CXR diagnosis or screening is to be performed based on the abnormality of CXR images. In fact, two national TB prevalence surveys in Cambodia [7, 38] showed that there were more TB cases with bacteriologically negative, but CXR suggestive of active TB, so called “minimal disease” [12] or “TB pathology” [39] in the community, than those with bacteriologically positive TB. Therefore, if we use a bacteriological reference only, the specificity becomes falsely low, and we cannot properly evaluate the CAD performance. In addition, we should consider the fact that there are falsely positive Xpert results among persons with past TB treatment history as well as falsely negative results below the lowest level of detection by Xpert because of the nature of polymerase chain reaction. Third, we compared F-CAD performance with only one fully experienced human reader in the study. However, the accuracy of the reader in this study was comparable to that of other readers in a study [20] with the bacteriological reference; a sensitivity/specificity with “abnormality suggestive of TB” was 84/78% (95%CI 78–90/75–80%) in this study and 89/63% (95%CI 87–90/62–63%) in that study, and a sensitivity/specificity with “any abnormality in the lung field” was 99/46% (95%CI 97–100/43–48%) and 95/46% (95%CI 94–96/45–46%), respectively.

In conclusion, AI–CAD is applicable to community-based ACF in high TB burden countries, where experienced human readers for CXR images are scarce. The study suggested that the use of AI–CAD in developing countries could expand CXR screening for TB for community-based ACFs with a substantial decrease in the workload on human readers and laboratory labour. Further studies are needed to generalize these results to other countries by increasing the participants being tested for bacteriological examination and comparing AI–CAD performance with that of more human readers.

## Data Availability

The data sets used and/or analyzed during the current study are available from the corresponding author on reasonable request.

## Abbreviations

AI–CAD: Artificial intelligence-based computer aided detection
ACF: Active case-finding
AUPRC: Area under the precision–recall curves (PRC)
AUROC: Area under the receiver operating characteristic (ROC) curves
95% CI: 95% Confidence interval
CXR: Chest radiography
HIV: Human immunodeficiency virus
IQR: Interquartile range
PCF: Passive case-finding
PPV: Positive predictive value
PRC: Precision–recall curve
ROC: Receiver operating characteristics
TB: Tuberculosis
TPP: Target product profile
